# Alcohol Metabolism’s Damaging Effects on the Cell: A Focus on Reactive Oxygen Generation by the Enzyme Cytochrome P450 2E1

**Published:** 2006

**Authors:** Dennis R. Koop

**Affiliations:** Dennis R. Koop, Ph.D., is a professor in the Department of Physiology and Pharmacology at Oregon Health and Science University, Portland, Oregon

**Keywords:** Ethanol metabolism, alcohol liver disorder, toxicity, mitochondria, alcohol dehydrogenase, acetaldehyde, cytochrome P450 2E1 (CYP2E1), oxidative stress, reactive oxygen species (ROS), superoxide

## Abstract

Alcohol metabolism’s various processes create harmful compounds that contribute to cell and tissue damage. In particular, the enzyme cytochrome P450 2E1 (CYP2E1) plays a role in creating a harmful condition known as oxidative stress. This condition is related to oxygen’s ability to accept electrons and the subsequent highly reactive and harmful byproducts created by these chemical reactions. CYP2E1’s use of oxygen in alcohol metabolism generates reactive oxygen species, ultimately leading to oxidative stress and tissue damage.

Alcohol is processed in the body through various metabolic pathways, producing toxic byproducts that contribute to cell and tissue damage. This article examines alcohol metabolism in the liver by the enzyme cytochrome P450 2E1 (CYP2E1) and the role of this enzyme in creating a harmful condition known as oxidative stress. (For an overview of the other metabolic pathways by which alcohol is broken down, see the article by Zakhari in this issue.) The toxicological significance of CYP2E1 was first appreciated when it was shown that this enzyme was responsible for the metabolism of many compounds to toxic products, and the toxicity was increased after synthesis of the enzyme was induced. In addition, researchers have found that CYP2E1 is associated with an increase in the reaction by which oxygen gains an electron (i.e., reduction of oxygen), which creates compounds referred to as reactive oxygen species (ROS), that can damage other cellular molecules. Furthermore, research with alcohol (i.e., ethanol)-treated animals has shown that CYP2E1 increases the chemical damage done by reactive molecules called free radicals to the lipid components of cell membranes (i.e., lipid peroxidation) in liver cells (Dey and Cederbaum 2006). Results from animal studies have shown a strong correlation between the level of CYP2E1 in the liver and the degree of alcohol-induced liver injury. That is, when the level of CYP2E1 is high there is more extensive lipid peroxidation, which is reduced by inhibiting CYP2EI induction. To understand how the expression of this enzyme can generate oxidative stress, it is useful first to understand how oxygen functions as an acceptor of electrons in the body and how P450 uses oxygen in alcohol metabolism.

## Oxidative Stress

**Figure 1:**
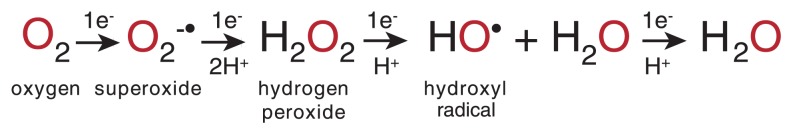
Sequential reduction of oxygen in four single-electron steps. The addition of one electron to oxygen (i.e., a one-electron reduction of oxygen) produces superoxide. If superoxide is reduced by another electron, the two-electron–reduced form of oxygen, peroxide, is produced. This will accept two hydrogens to produce hydrogen peroxide. Hydrogen peroxide can accept another single electron, and this usually occurs as a result of the transfer from a reduced metal ion (e.g., reduced iron or copper). When it does accept that electron, hydrogen peroxide is converted to a hydroxyl radical (HO^•^) and a hydroxide anion (HO^−^) that, when combined with hydrogen, produces water.

Oxidative stress occurs in the presence of an excess of reactive molecules called free radicals or a lack of molecules that can eliminate free radicals (i.e., antioxidants). Free radicals are highly reactive molecules that interact with other cellular structures. They contain unpaired electrons and therefore seek to obtain other electrons so that a stable pair is produced. For example, oxygen has two unpaired electrons. As the ultimate electron acceptor in cellular metabolism for organisms that use oxygen (i.e., aerobic organisms), oxygen can accept four electrons before becoming a neutral molecule (i.e., it can be reduced by four electrons). The intermediate steps to give the final four-electron–reduced molecule produces ROS. Figure 1 shows this process and its products. An increase in free radicals usually is the result of an increased reduction of oxygen to ROS, which can react with other cellular molecules. Recent reviews (Lieber 2004; Gonzalez 2005; Dey and Cederbaum 2006) have examined the mechanisms of alcohol-induced damage resulting from the generation of these reactive species.

### Superoxide

The addition of one electron to oxygen (i.e., a one-electron reduction of oxygen) produces superoxide, abbreviated O_2_^−•^. The dot indicates a radical species, and superoxide is usually shown with a negative sign, indicating that it carries a charge of negative one as a result of the one-electron reduction. Superoxide can either accept an electron (i.e., it can act as an oxidant) or it can donate the extra electron to another molecule (i.e., it can act as a reductant). Superoxide radicals are formed most often in the part of the cell called the mitochondria during normal cellular energy-producing processes and, to a much lower extent, in the fluid of the cell (i.e., cell cytosol). Cells also have enzymes called superoxide dismutases. These enzymes are among the first line of defense in the detoxification of products resulting from oxidative stress. They convert superoxide radicals to hydrogen peroxide and protect the cell from high concentrations of this one-electron–reduced form of oxygen. One form of the enzyme, a copper/zinc (Cu/Zn) superoxide dis-mutase, is present in the cytosol; manganese (Mn) superoxide dismutase is present in the mitochondria. If the concentration of superoxide becomes too high, it can participate in the formation of the highly reactive hydroxyl radical (HO^•^). Superoxide does this by donating the extra electron to metal ions (for example, ferric iron or Fe^3+^) that then can act as the catalyst to convert hydrogen peroxide into the hydroxyl radical (HO^•^).

### Hydrogen Peroxide and the Hydroxyl Radical

If superoxide is reduced by one electron, the two-electron–reduced form of oxygen, peroxide (O_2_^−2^), is produced. The charge of negative two reflects the additional two electrons that were accepted by oxygen. This negatively charged species will easily accept two hydrogens to produce hydrogen peroxide (H_2_O_2_). Now, being a neutral (i.e., uncharged) molecule, hydrogen peroxide can pass readily through cell membranes and cannot be excluded from cells. The cell produces certain enzymes (glutathione peroxidase and catalase) that guard against increased concentrations of hydrogen peroxide, in both the cytosol and mitochondrial portions of the cell.

Hydrogen peroxide is not completely inactive. It can accept another single electron, and this usually occurs as a result of the transfer from a reduced metal ion (e.g., reduced iron or copper). When it does accept that electron, hydrogen peroxide is converted to a hydroxyl radical (HO^•^) and a hydroxide anion (HO^−^) that, when combined with hydrogen, produces water. The hydroxyl radical is extremely reactive and will take an electron from the first molecule that it touches. The reaction generates radical species that then propagate free-radical chain reactions. These chain reactions destroy cell membranes and damage DNA, RNA, and proteins, resulting in damage to cells and organs. When a hydroxyl radical takes the last electron from other cellular molecules, it still has a negative charge that will accept a proton, producing water. Thus, if no side reactions were to occur and oxygen was reduced by four electrons, this would generate two molecules of water, one from each oxygen atom in the original oxygen molecule, as shown in Figure 1. In a perfect world, no side reactions would take place and this breakdown would occur. However, the fact that the cell has enzymes to protect against the generation of intermediate reduced forms of oxygen indicates that these side reactions do occur, and, indeed, they occur more readily after alcohol consumption.

## Alcohol Oxidation and the Generation of Reactive Oxygen Species

Alcohol is metabolized by oxidation; that is, it loses electrons. The cell oxidizes alcohol first to acetaldehyde (via the enzyme alcohol dehydrogenase) and then oxidizes acetaldehyde to acetic acid (via the enzyme acetaldehyde dehydrogenase). These reactions involve an intermediate carrier of electrons, nicotinamide adenine dinucleotide (NAD), which is reduced by two electrons to form NADH. The electrons that originated in alcohol to produce NADH ultimately are transferred to oxygen via a number of other intermediate carriers in the mitochondrial electron transport chain, and oxygen is reduced to water at the terminal enzyme (cytochrome oxidase) of that chain. It is the movement of the electrons down this enzyme chain in the mitochondria that accounts for the fact that alcohol is a significant source of energy (Lieber 2005).

The delivery of electrons to oxygen must be very carefully controlled to prevent the generation of the intermediate ROS described above. A small, but significant, portion of the oxygen that is reduced in the mitochondria is released as the one-electron–reduced form, superoxide. Thus, increased mitochondrial activity and NADH use will result in greater superoxide production. As described above, the mitochondria are protected against high levels of superoxide by the enzyme manganese superoxide dismutase.

### Alcohol Oxidation by P450

In addition to the well-regulated oxidation of alcohol by alcohol dehydrogenase, another oxidation pathway involving cytochrome P450 is present predominately in liver tissue. Although the P450 pathway contributes a small amount to total alcohol oxidation (Lands 1998), the generation of ROS is significant as a result of the catalytic mechanism of the enzyme and is important to the generation of oxidative stress associated with alcohol metabolism. The P450dependent oxidation of alcohol was first identified as the microsomal ethanol oxidizing system (MEOS) and was shown to involve the activity of primarily one form of P450, now known as CYP2E1 (Koop and Coon 1986; Lieber 2004).

**Naming and Categorizing the P450 Enzymes**

Cytochrome P450 (P450 or CYP) is a term used to describe a large number of enzymes that are common in all species. P450 has been found in the DNA sequences of a variety of plants and animals. DNA sequences ultimately code for all the proteins in the cell. By examining the different DNA sequences, researchers have been able to identify other enzymes similar to P450.

This type of analysis also has revealed the complexity of the P450 enzyme family (Nelson 1998). The number of different P450s in a given species varies considerably. For example, the mouse DNA sequence suggests the presence of 102 functional P450s, whereas humans have 57 functional P450s (Nelson et al. 2004).

To track the different P450s identified by examining DNA gene sequences, researchers have developed a systematic nomenclature system.^1^ The DNA provides the information that defines the linear sequence of amino acids that comprise the sequence of a protein (that is, knowing the sequence of the gene enables researchers to predict the sequence of the protein). Each protein has a unique sequence of amino acids). The nomenclature for the P450s involves comparing the linear sequence of amino acids that make up the P450s. Modern computer programs allow researchers to compare the amino acid sequences of all P450s predicted from the DNA sequences.

Although somewhat arbitrary, those P450s that have 40 percent of their amino acids in the same linear sequence are placed into the same “family,” which is designated by a number following CYP. Thus, the alcohol-inducible form of cytochrome P450 is in the CYP2 family of P450s. A further comparison is made, and if the amino acids in the same family are identical 55 percent of the time, the two enzymes are in the same subfamily, which is designated by a letter following the family, thus CYP2E. Then each individual enzyme in the subfamily is given a number (i.e., CYP2E1). Because one member of the 2E subfamily was found in all species examined, the CYP2E family has one enzyme, CYP2E1. (The rabbit has a second enzyme, named CYP2E2.) Most other subfamilies have more than one member in a given species. For example, in humans, the CYP2C family has four members, called CYP2C8, CYP2C9, CYP2C18, and CYP2C19.

ReferencesNelsonDRCytochrome P450 nomenclatureMethods in Molecular Biology107152419981457720910.1385/0-89603-519-0:15NelsonDRZeldinDCHoffmanSMComparison of cytochrome P450 (CYP) genes from the mouse and human genomes, including nomenclature recommendations for genes, pseudogenes and alternative-splice variantsPharmacogenetics1411820041512804610.1097/00008571-200401000-00001

**Figure 2:**
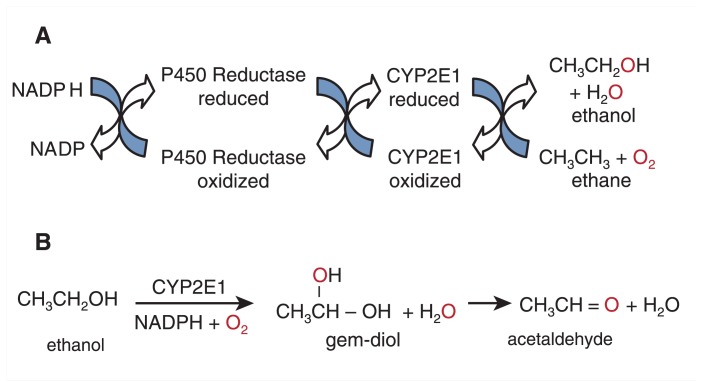
Metabolism of ethane to produce alcohol and water. Electrons are transferred from NADPH to cytochrome P450 2E1 (CYP2E1) by cytochrome P450 reductase. **A**) The CYP2E1 then catalyzes the oxidation of the substrate molecule (ethane) by adding one atom of oxygen to the substrate, producing ethanol; the other atom is reduced to water. **B**) The CYP2E1catalyzed metabolism of alcohol produces an unstable intermediate (i.e., a gem-diol) that will decompose to produce acetaldehyde.

The P450s are heme proteins, meaning they consist of amino acids that fold in a way that they surround a nonprotein component that contains iron (i.e., a heme prosthetic group). They have a cysteine amino acid side chain that binds to the iron of the heme. This characteristic differentiates P450 from other heme-containing proteins, such as the cytochromes of the electron transport chain in the mitochondria, and hemoglobin, the oxygen carrier in blood. A given species may have many different P450s (see Sidebar), and the reason for this is not completely understood. What is clear is that this unique amino acid binding to the heme allows P450 to catalyze the metabolism of a wide variety of substances (i.e., substrates) that originate both inside (i.e., endogenous) and outside (i.e., exogenous) the organism. Many of the P450s are essential for making steroids and other molecules like cholesterol. Others have evolved to metabolize compounds that come from the environment. The reaction catalyzed by all of the P450s is the same; it is a reaction where one atom of an oxygen molecule is added to a substrate molecule and the other atom is reduced to water. Figure 2*A* shows this reaction for ethane, a simple substrate molecule for CYP2E1. This molecule is metabolized (oxidized in this case) by the addition of one oxygen atom to the ethane to produce alcohol, and the other atom in the oxygen molecule is reduced to water. This reaction occurs in a network of membranes within the cell known as the endoplasmic reticulum and involves a second protein, called P450 reductase. This protein transfers electrons one at a time to the CYP2E1 heme iron after first accepting them from the reduced coenzyme nicotinamide adenine dinucleotide phosphate (NADPH). Thus, the essence of the reaction catalyzed by all the P450s is essentially the same, but the nature of the substrate differs. Of the 57 P450s in humans, 15 are involved in the metabolism of exogenous drugs and chemicals (Guengerich 2006). CYP2E1 is in this group and can catalyze the oxidation of many small organic compounds. For example, CYP2E1 might have an important role in producing glucose molecules from ketones such as acetone and acetol during starvation (Koop 1992; Guengerich 2003).

The CYP2E1-dependent metabolism of alcohol produces an initial product, the gem-diol, which is chemically unstable and decomposes to acetaldehyde, as illustrated in Figure 2*B*. Thus, the same product that is produced by alcohol dehydrogenase is formed by CYP2E1, but the mechanism is very different. It is this mechanism of using oxygen to metabolize alcohol that can lead to the generation of ROS. As a result of generating ROS, CYP2E1 also will indirectly catalyze the formation of a radical species from ethanol itself (1hydroxyethyl radical), which also contributes to oxidative damage.

**Figure 3:**
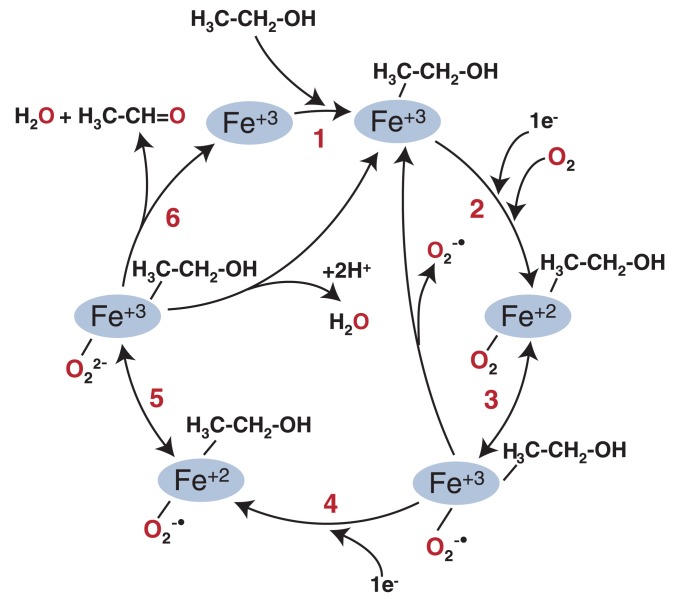
Generation of reactive oxygen by cytochrome P450 2E1 (CYP2E1). CYP2E1 is represented by the heme iron in the blue ovals. When CYP2E1 uses oxygen to metabolize alcohol, reactive oxygen species (ROS) can be generated by the following chain of events: ethanol binds to the enzyme (step 1). As the first electron is passed to the heme of CYP2E1 and oxygen is bound (step 2), the electron can move and exist on the oxygen, essentially generating superoxide bound to the heme of CYP2E1 (step 3). Occasionally, the superoxide will break down, releasing free superoxide and generating the starting enzyme. If the second electron is added to the enzyme (step 4), then a second form of reduced oxygen is produced that is identical to a heme-bound form of the twoelectron–reduced oxygen (i.e., peroxide) (step 5). When this product breaks down, it picks up two hydrogens to generate hydrogen peroxide. The production of these ROS by CYP2E1 is referred to as an “uncoupled reaction” because the oxygen does not end up in the substrate. If the reduced oxygen species remains bound, then the enzyme will transfer one oxygen atom to the substrate and the other atom becomes water, producing an unstable intermediate (i.e., a gem-diol) product that decomposes to acetaldehyde (step 6).

A very important feature of CYP2E1 (and of other P450s as well) is that when the enzyme uses oxygen in the reaction, ultimately adding one atom of oxygen to the substrate molecule, sometimes the reaction does not proceed as planned and the enzyme itself can generate the ROS described above. This is illustrated in Figure 3, which shows alcohol as the molecule being broken down by the enzyme, although it also has been demonstrated that this reaction can occur in the absence of any substrate. The sequence of events that occurs and allows CYP2E1 to catalyze the metabolism of alcohol essentially is identical to those steps described above for the reduction of oxygen in single-electron steps. As the first electron is passed to the heme of CYP2E1 and oxygen is bound (Figure 3, step 2), the electron can move and exist on the oxygen, essentially generating superoxide bound to the heme of CYP2E1 (Figure 3, step 3). Occasionally, the complex will break down, releasing free superoxide and generating the starting enzyme. If the second electron is added to the enzyme, then a second form of reduced oxygen is produced that is identical to a heme-bound form of the two-electron–reduced oxygen (i.e., peroxide), as shown in Figure 3, steps 4 and 5. When this complex breaks down, it picks up two hydrogens to generate hydrogen peroxide. The production of these ROS by CYP2E1 is referred to as an “uncoupled reaction” because the oxygen does not end up in the substrate. If the reduced oxygen species remains bound, then the enzyme will transfer one oxygen atom to the substrate and the other atom becomes water (Figure 3, step 6).

The generation of these ROS by CYP2E1 contributes to the oxidative stress observed after alcohol consumption. CYP2E1 is unique in that it is 1 of 57 different P450s found in humans that will readily accept electrons and generate these ROS even in the absence of a substrate. Most P450s will not accept electrons unless the substrate is first bound to the enzyme. After binding a substrate, other P450s also will generate some reactive oxygen. The extent of this reaction is dependent on the substrate. CYP2E1 appears to be more “uncoupled” than other P450s, meaning that it generates ROS and oxidative stress much more readily than other P450s. When alcohol is consumed, CYP2E1 also will generate acetaldehyde as well as ROS in the cytosol of the cell, leading to an increase in oxidative stress. In addition to acetaldehyde, the ROS produced by CYP2E1 also can result in the formation of the reactive 1-hydroxyethyl radical, which also contributes to alcohol-induced oxidative stress.

## Varying Levels of CYP2E1 in the Body

**Figure 4:**
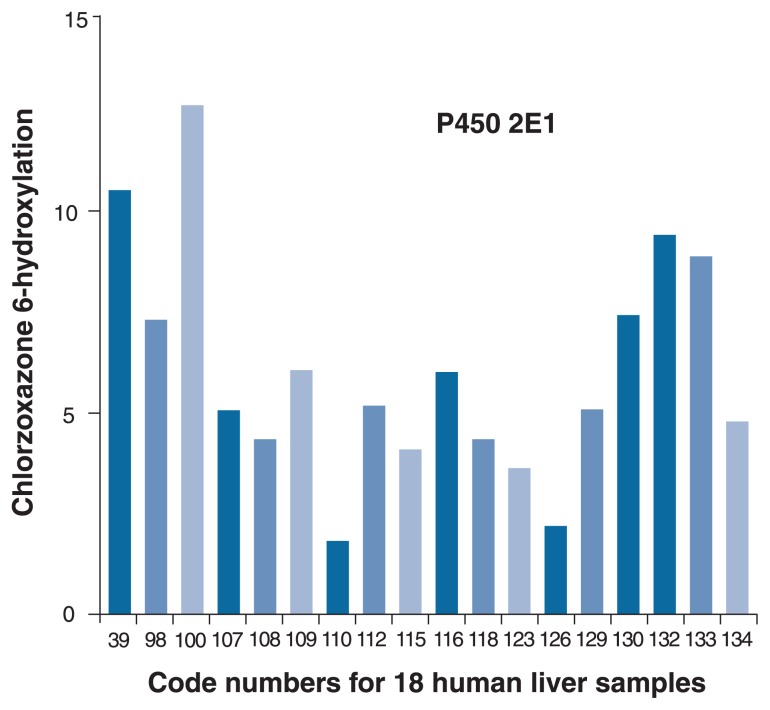
The variability of cytochrome P450 2E1 (CYP2E1) from 18 human liver samples. Each liver sample is designated with a code number. The level of the of the enzyme is directly proportional to the formation of 6-hydyroxychlorzoxazone. Source: Guengerich 2006.

The amount of CYP2E1 in the liver is extremely variable, as shown in Figure 4. This figure shows the amount of the enzyme found in liver tissue samples, as revealed by monitoring the metabolism of the drug chlorzoxazone, which is predominately metabolized by CYP2E1. The difference in CYP2E1 levels observed in just the 18 human liver samples shown in Figure 4 is approximately eight-fold. Importantly, it is well established that alcohol consumption will increase the concentration of CYP2E1 in the liver as well as other tissues (Lieber 2004). For example, Oneta and colleagues (2002) conducted a controlled trial that showed that the consumption of 40 grams^1^ of alcohol per day for 4 weeks resulted in about a five-fold increase in the level of CYP2E1, as determined by chlorzoxazone metabolism. It is expected that under these conditions there also would be a concomitant increase in the generation of reactive species from the enzyme. In addition, Gonzalez (2005) suggested that other P450s also may contribute to the generation of oxidative stress. This is based on research with mouse models where specific P450s were deleted from the mouse gene. Whether this is true in humans remains to be determined.

Compounds other than alcohol also are very good inducers of CYP2E1, including 4-methylpyrazole, a drug now used for the treatment of methanol and ethylene glycol poisoning. It is of interest because this drug also is a very potent inhibitor of alcohol dehydrogenase. The mechanism for regulating the enzyme concentration is complex and is linked to the metabolic state. For example, the level of CYP2E1 is lower during times when the body has received adequate nutrition (i.e., the fed state) and higher during times of starvation or with disease states such as obesity. Many different mechanisms are involved in the regulation of enzymes, such as controlling how much RNA is produced from the DNA, how the RNA is translated into the protein, and the degradation of the enzyme after it is produced. Many endogenous and exogenous compounds are substrates for CYP2E1 and can stabilize the enzyme and thus increase the concentration (Ingelman-Sundberg 2004; Rodriguez-Antona and Ingelman-Sundberg 2006). Thus, the level of CYP2E1 is under complex regulatory control, and factors in addition to alcohol consumption, such as the nutritional state, environmental exposures, obesity, diabetes, and liver disease can contribute to the large variation of P450 found in the population.

Researchers have identified several genetic variations (i.e., polymorphisms) in the human CYP2E1 gene 2 (Ingelman-Sundberg 2004). Several studies have attempted to link the polymorphisms to the incidence of different types of cancers, alcoholic liver disease, and alcoholism (Ingelman-Sundberg and Rodriguez-Antona 2005). Unfortunately, there are little, if any, data that link the polymorphisms reported to changes in CYP2E1 activity. The most important factor in the level and thus the activity of the enzyme is probably how easily the enzyme is induced by alcohol itself. It has been demonstrated clearly that increased alcohol consumption results in increased expression of CYP2E1, which is correlated with liver injury in animal models. Increased expression of CYP2E1 in humans consuming alcohol also has been reported (Takahashi et al. 1993), thus implicating CYP2E1 in human alcohol-induced liver injury.

## Conclusion

Animals have evolved to express a large number of P450s that are essential for the production of many hormones and sterols which are essential for regulating basic physiological function and cell structure. In addition, these enzymes are responsible for the metabolism of exogenous compounds. Alcohol is one of many exogenous compounds that is consumed orally and which can be metabolized by P450, especially the form CYP2E1. Although the contribution of this enzyme to total alcohol metabolism is small, it plays a very significant role in the generation of ROS as well as of oxidative stress. More importantly, the presence of alcohol can increase the level of the enzyme, which in turn results in the generation of more ROS, exacerbating the potential for cell and tissue damage. Understanding CYP2E1’s role in alcohol metabolism and the generation of ROS is therefore important to ultimately understanding alcohol-related tissue damage.

## Footnotes

1 A summary of the nomenclature for P450 enzyme family can be found on the home page of Dr. David Nelson (available at: http://drnelson.utmem.edu/matrix.html). This Web site also has links to other useful P450 databases.

1 A standard drink is defined as 12 fluid ounces of beer, 5 fluid ounces of wine, or 1.5 fluid ounces of distilled sprits and contains approximately 0.5 ounces (i.e., 14 grams) of alcohol. Thus, three standard drinks would equal 42 grams of alcohol.

2 As with the naming of the P450s (see Sidebar), a systematic nomenclature has been adopted for the nomenclature for polymorphic P450s. A Web page describing all of the current polymorphism for CYP2E1 is available at http://www.imm.ki.se/cypalleles/CYP2E1.htm.
